# *Shigella dysenteriae* Serotype 1, Kolkata, India

**DOI:** 10.3201/eid0911.020652

**Published:** 2003-11

**Authors:** Shanta Dutta, Dharitri Dutta, Phalguni Dutta, Shigeru Matsushita, Sujit Kumar Bhattacharya, Shin-ichi Yoshida

**Affiliations:** * National Institute of Cholera and Enteric Diseases, Kolkata, India; † Metropolitan Research Laboratory of Public Health, Tokyo, Japan; ‡ Faculty of Medical Sciences, Kyushu University, Fukuoka, Japan

**Keywords:** shigellosis, *S. dysenteriae* 1, drug resistance

## Abstract

Since July 2002, bacteriologically confirmed shigellosis cases have increased, and multidrug-resistant *Shigella dysenteriae* serotype 1 strains have reemerged in patients hospitalized with diarrhea in Kolkata, India. The isolated strains of *S. dysenteriae* 1 showed resistance to chloramphenicol (80%), ampicillin (100%), tetracycline (100%), co-trimoxazole (100%), nalidixic acid (100%), norfloxacin (100%), and ciprofloxacin (100%). Emergence of fluoroquinolone resistance in *S. dysenteriae* 1 strains complicated treatment of shigellosis patients. Six strains belonging to provisional serovars of *S. dysenteriae* were also identified for the first time in patients hospitalized with diarrhea in Kolkata, India.

Shigellosis is an important cause of bloody diarrhea in all age groups, especially in children. Of all serotypes of shigellae, *Shigella dysenteriae* type 1 attracts special attention for its epidemic-causing potential and its association with most serious dysentery cases, with a high attack rate, high case-fatality rate, and various complications (*1*). Antimicrobial therapy is usually recommended for treatment of shigellosis. However, antimicrobial resistance in enteric pathogens, including *Shigella* isolates, complicates the situation in developing countries, where shigellosis is endemic and indiscriminate use of antimicrobial agents is common.

During early 1984, various areas of India, including the eastern region, witnessed an extensive epidemic of bloody dysentery, predominantly caused by multidrug-resistant *S. dysenteriae* type 1, which swept through the districts of West Bengal from north to south. The strains were resistant to streptomycin, tetracycline, and chloramphenicol; highly sensitive to nalidixic acid, gentamicin, furazolidone; and moderately sensitive to ampicillin and cotrimoxazole (*2*). Nalidixic acid–resistant strains of *S. dysenteriae* 1 emerged in Eastern India during 1988 (*3*). In 1992, *S. dysenteriae* 1 was isolated from 24% of total bloody diarrhea case-patients, and the strain showed resistance to nalidixic acid (30%), furazolidone (2%), ampicillin (95%), and co-trimoxazole (88%). All strains were susceptible to fluoroquinolone derivatives, i.e., norfloxacin and ciprofloxacin (*4*). Therefore, furazoldione and nalidixic acid were used as first-line drugs for shigellosis during that period, with selective use of fluoroquinolones.

Changes in the worldwide epidemiology of shigellae species have been documented in the last two decades**.** Although bacteriologically confirmed childhood shigellosis cases varied from 4% to 6%, a change in serotypes and antimicrobial resistance in *Shigella* species was noticed in Kolkata during 1995–2000 (*5*). *S. flexneri* (58%) completely replaced *S. dysenteriae* (5%) and became the most prevalent serotype, followed by *S. sonnei* (28%) and *S. boydii* (9%). During 1997 to 2000; *S. dysenteriae* type 1 strain was not isolated. One strain of *S. dysenteriae,* isolated in 1999, and three strains of *S. dysenteriae,* isolated in 2000, belonged to *S. dysenteriae* type 2 (unpub. data). Isolated strains were resistant to nalidixic acid (29% with MIC_90_ <128 μg/mL), tetracycline (90%), co-trimoxazole (90%), ampicillin (67%), and chloramphenicol (46%). Again all strains were susceptible to norfloxacin (MIC_90_ <1 μg/mL) and ciprofloxacin (MIC_90_ = 0.125 μg/mL), rendering them drugs of choice for treatment of shigellosis in recent years. Routine surveillance data from National Institute of Cholera and Enteric Diseases (NICED) showed a 1% to 2% isolation rate of all *Shigella* serotypes from diarrhea patients since 1997, with the identification of a single strain of *S. dysenteriae* type 1 in 1998.

This study, performed as a continuation of routine surveillance for diarrheal diseases in two large hospitals in Kolkata, found a recent increase in patients seeking treatment for acute and severe bloody diarrhea and the reemergence of *S. dysenteriae* 1 strains with altered antibiogram.

## The Study

During April–May 2002, an outbreak of bacillary dysentery was reported in the northern district of West Bengal, India, among tea garden workers. A team from National Institute of Cholera and Enteric Diseases investigated the episode, and *S. dysenteriae* 1 was found to be the sole causative agent of the outbreak (*6*). A similar outbreak of blood dysentery caused by *S. dysenteriae* 1 occurred during March–June 2002 in the southern part of West Bengal (*7*). Following these episodes, we intensified the surveillance of diarrheal diseases in two hospitals of Kolkata, India. Infectious Disease (I.D.) Hospital is the biggest hospital in Kolkata, if not in India, for admission and treatment of infectious disease cases and the Dr. B. C. Roy Memorial Children’s Hospital is the only referral pediatric hospital in the state of West Bengal, which usually serves an area that includes the Kolkata metropolis and suburbs. In both hospitals, patients with diarrhea were kept under continuous surveillance after admission to the Diarrhoea Treatment Unit (DTU) of the hospitals and were treated with oral rehydration solution and antimicrobial agents as advised by attending clinicians.

Rectal swabs or fresh stool samples were collected from children with acute diarrhea admitted to Dr. B. C. Roy Memorial Children’s Hospital, Kolkata from January 2001 through August 2002. The children were selected irrespective of type, duration of diarrhea, and history of antibiotic drug therapy.

During the first week of July 2002, an upsurge of acute bloody diarrhea cases was noticed in patients attending the I. D. Hospital, Kolkata, and increased numbers of patients with acute dysentery continued to be admitted to the hospital until September 2002.The patients reported bloody stools, abdominal pain, and tenesmus, with or without fever. Stool samples or rectal swabs were collected from all of these patients on admission from July 1, 2002, to August 31, 2002.

The samples were placed in Cary-Blair transport medium and processed within 2 hours of collection in the Microbiology Laboratory of the National Institute of Cholera and Enteric Diseases, Kolkata; the samples were tested for the entire gamut of enteropathogens by using standard techniques. *Shigella* species were confirmed with the API 20E test (Biomerieux, Marcy l’Etoile, France) and slide agglutination test with antisera specific to serotypes of *Shigella* species (Denka Seiken Co, Tokyo, Japan). Antimicrobial susceptibility testing of *Shigella* isolates was done by the disk diffusion method, and MICs were measured by using the agar dilution method.

In addition to the conventional technique, polymerase chain reaction (PCR) was performed within 4–6 hours on LB broth cultures of 77 stool samples collected from I. D. Hospital by using published primer sequences for *Ipa*H (invasion plasmid antigen H) gene to detect further *Shigella* infection, which might have been missed by conventional methods (*8*). PCR was also performed in anticipation of rapid diagnosis and early treatment of shigellosis cases and thus prevent the development of complications.

Table 1 shows the distribution of *Shigella* serotypes from patients admitted in two hospitals of Kolkata. An increased isolation rate of all serotypes of shigellae was observed in patients with acute diarrhea (72/790; 9.1%) and also in patients with bloody diarrhea (72/237; 30%) in the Children’s Hospital since January 2002. Although *S. flexneri* continued to be the most prevalent serotype (45/72; 62.5%), followed by *S. sonnei* (18/72; 25%), the reemergence of *S. dysenteriae* serotype 1 (5/72; 7%) claimed special attention. During 2001, only one strain of *S. dysenteriae* 1 was isolated in July, but during January to August 2002, five *S.* *dysenteriae* 1 strains were identified:one in April and two in both July and August 2002. The other four *S. dysenteriae* strains isolated in 2001 were *S. dysenteriae* type 2 (two strains), *S.* *dysenteriae* type 3 (one strain), and *S. dysenteriae* type 6 (one strain).

**Table 1:** Isolation frequency of *Shigella* serotypes from patients admitted to two different hospitals, Kolkata, India

Place, y, and period of sample collection	No. samples tested (n)	Strains *Shigella* isolated n (%)	*S. dysenteriae* (n)	*S. flexneri* (n)	*S. boydii* (n)	*S. sonnei* (n)
**B.C. Roy Memorial** **Children’s Hospital**						
2001 Jan–April May–Aug Sept–Dec 2002 Jan–April May–Aug	1,069 442 397 230 790 365 425	80 (7.5) 26 (5.9) 39 (9.8) 15 (6.5) 72 (9.1) 32 (8.7) 40 (9.4)	5 1 4 0 5 1 4	40 13 17 10 45 26 19	9 3 5 1 4 1 3	26 9 13 4 18 4 14
**Infectious Diseases** **Hospital**						
2002 July–Aug	77	27 (35)	24	2	0	1

Among 77 patients with bloody diarrhea in I. D. Hospital examined through August 2002, *Shigella* spp. were identified as the sole pathogen from 27 (35%) patients. Of 27 *Shigella* strains, 24 (88%) belonged to *S. dysenteriae* serotype 1; other strains isolated were *S. flexneri* (2 strains) and *S. sonnei* (1 strain). PCR could detect shigellae infection in 37 (48%) patients, and 12 of these patients were not culture-positive. No other pathogen could be detected from any of the case-patients. The figure shows the epidemic curve for *S. dysenteriae* 1 case-patients admitted to I. D. Hospital during the period of study.

**Figure:**
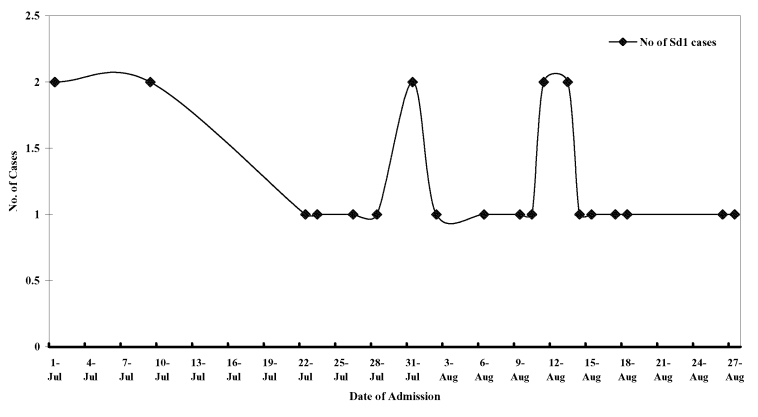
Epidemic curve for *Shigella dysenteriae* 1 case-patients admitted to Infectious Diseases Hospital, Kolkata, India, during July and August 2002.

The antimicrobial resistance profiles of the isolated *S. dysenteriae* 1 strains showed multidrug resistance to at least seven or more antimicrobial agents, e.g., chloramphenicol (80%), ampicillin (100%), tetracycline (100%), cotrimoxazole (100%), furazolidone (50%), nalidixic acid (100%), ciprofloxacin (100%), norfloxacin (100%), and amoxicillin (100%). MICs of antimicrobial agents also showed higher level of resistance acquired by these strains compared to the MICs observed during 1995–2000 (*5*). The MICs_90_ of antimicrobial agents tested were as follows: ampicillin (512 μg/mL), tetracycline (>256 μg/ml), chloramphenicol (<256 μg/mL), nalidixic acid (>256 μg/mL), norfloxacin (>32 μg/mL), and ciprofloxacin (>8 μg/mL). In contrast, other serotypes of shigellae were uniformly susceptible (100%) to ciprofloxacin and norfloxacin and showed partial resistance to ampicillin (60%), tetracycline (94%), cotrimoxazole (98%), and nalidixic acid (45%).

Initially, physicians advised norfloxacin and ciprofloxacin for routine treatment of shigellosis, because of poor clinical responses, subsequent patients were treated with ofloxacin to which the organism was susceptible (100%). No case fatality and no case of hemolytic uremic syndrome have been reported among the present series of patients. Median time for resolution of symptoms of the patients was 4 days from the date of admission to the hospital.

When *S. dysenteriae* 1 strains were screened for virulent gene profiles by PCR with published primer sequences (*9*), all *S. dysenteriae* 1 strains (100%) were found to harbor *stx*1 (Shiga toxin), *ipa*H (invasion plasmid antigen H), and *ial* (invasion-associated locus) gene and were negative for *set* (*Shigella* enterotoxin 1) gene. Sixteen (70%) of 23 isolates were positive for *sen* (*Shigella* enterotoxin 2) gene. Only one *S. dysenteriae* 1 strain showed both *Shigella* enterotoxin 1 and 2 (*set* and *sen*) genes.

Although the proportion of *Shigella* strains isolated from case-patients with acute bloody diarrhea increased (30% to 35%), the rate of *S. dysenteriae* 1 isolated from B.C. Roy Memorial Children’s Hospital (5/237; 2%) was not as high as that of patients at I.D. Hospital (27/77; 35%). Because I. D. Hospital was a general hospital and provided treatment to patients of all ages, including children, concerned parents of patients who had severe dysentery caused by *S. dysenteriae* 1 may have brought them to I.D. Hospital in anticipation that their illness would be better managed.

Isolated strains of *S. dysenteriae* 1 from two recently documented outbreaks in West Bengal also showed reduced susceptibility to fluoroquinolones (*6*,*7*). In recent years, the emergence of multidrug-resistant *S. dysenteriae* 1 strains has also been reported from Southeast Asia and Africa, although fluoroquinolone resistance was not observed among the strains (*10*,*11*).

While processing the stool samples of patients admitted to the Children’s Hospital for shigellae species by conventional method, we found a few strains that showed a biochemical reaction typical of *Shigella*, but were nonagglutinable by commercially available antisera. These strains were positive for *ipa*H gene when tested by PCR. We designated those strains as *Shigella* untypable strains (*8*). The strains were sent to the Tokyo Metropolitan Research Laboratory of Public Health, Tokyo, Japan, for typing, and they were found to be provisional serovers of *Shigella* spp. (Table 2). Table 2 shows the year of isolation and virulent gene profiles of those strains. All strains were negative for *stx*1 gene. They were resistant to chloramphenicol, tetracycline, cotrimoxazole, furazolidone, and amoxicillin. But all strains were susceptible to nalidixic acid, norfloxacin, ciprofloxacin, gentamicin, amikacin, and cefotaxime. This drug-resistance profile contrasted with that of recently emerged *S. dysenteriae* type 1 strains. Similar strains have also been identified in other studies (*12*–*14*). To our knowledge, this is the first report of the isolation and identification of provisional serovars of *S. dysenteriae* and *S. boydii* from Kolkata, India.

**Table 2:** Virulence gene profiles of provisional serovars of *Shigella* spp. isolated from Kolkata, India

Serial no.	New serovars *Shigella* spp. (n)	Mo and y of isolation	Detection of virulence genes by PCR^a^				
			*Ipa*H	*ial*	*set*	*sen*	*stx*1
1. 2. 3. 4. 5. 6. 7.	*S. dysenteriae* 204/96 *S. boydii* E16553 *S. dysenteriae* 93-119 *S. dysenteriae* 204/96 *S. dysenteriae* E-23507 *S. dysenteriae* I-9809-93 *S. dysenteriae* 204/96	May 2000 June 2000 Oct 2000 Aug 2001 Aug 2001 Dec 2001 Mar 2002	+ + + + + + +	+ + + + + + +	- - - - - - -	+ + + + + - +	- - - - - - -

^a^PCR, polymerase chain reaction; +, positive; -, negative

## Conclusions

Our study reports increased isolation of shigellae with reemergence of *S. dysenteriae* 1 in and around Kolkata, India. This increase has public health importance with respect to monitoring impending outbreaks of shigellosis and implementing appropriate strategies for containment of this deadly organism.

Emergence of multidrug-resistant *Shigella* strains is of concern to clinicians in treating shigellosis cases. Because the recently emerged *S. dysenteriae* 1 strain was resistant (100%) to ampicillin, cotrimoxazole, nalidixic acid, norfloxacin, and ciprofloxacin, which were commonly used for shigellosis cases; ofloxacin is currently recommended for treatment. Concomitant search for alternate new drugs should be continued because, although newer antimicrobial drugs can offer hope for treatment of shigellosis, emergence of resistance to the new drugs is also not far in the future. Therefore, generating an effective vaccine can offer the ultimate solution to such problems. Perhaps the more effective way of reducing the impact of the disease and the risk of contracting infection lies in improving poor living condition, disseminating health education, and supplying safe drinking water. However, accomplishing those objectives and reaching the goal is not an easy task in developing countries. Laboratory detection capabilities also need to be strengthened at all levels to increase the baseline surveillance data for improved isolation of the pathogen. Identifying some strains with provisional serovars of *Shigella* spp. for the first time from Kolkata, India, indicates that all provisionally identified *Shigella* strains should be sent to a reference laboratory for typing and further characterization.

Studying plasmid profiles of isolated *S. dysenteriae* 1 strains and typing the strains by using various molecular tools could provide insight into the origin of these recently isolated *S.* *dysenteriae* 1 strains and the relationships among the strains.
